# Does Pronator Quadratus Repair Affect the Functional Outcome Following Volar Plate Fixation for Distal End Radius Fracture?

**DOI:** 10.7759/cureus.103695

**Published:** 2026-02-16

**Authors:** Kashyap Ravishankar, Vinod Kumar K, Arun Kumaar, Arun H Shanthappa

**Affiliations:** 1 Department of Orthopedics, Sri Devaraj Urs Medical College, Kolar, IND

**Keywords:** bone plates, distal radius fractures, pronator quadratus, recovery of function, wrist joint

## Abstract

Background and objective

Distal radius fractures represent the most common upper extremity injury requiring surgical intervention. Volar plate fixation necessitates division of the pronator quadratus muscle, yet the necessity of systematic muscle repair remains controversial. While some studies suggest enhanced functional outcomes with repair, others demonstrate equivalent recovery patterns regardless of muscle reconstruction. This study aimed to analyze functional outcomes following volar plate fixation for distal radius fractures, comparing patients with and without pronator quadratus repair.

Methodology

A retrospective cohort study was conducted involving sixty patients with displaced distal radius fractures treated with volar plate fixation between September 2023 and October 2024. Patients were stratified into pronator quadratus repair (n=31) and non-repair (n=29) groups. Functional assessment utilized the validated Patient-Rated Wrist Evaluation questionnaire at eight weeks, three months, and six months postoperatively. Additional measurements included range of motion parameters, grip strength assessment using standardized dynamometry, and isokinetic forearm rotation strength evaluation. Statistical analysis employed independent t-tests, chi-square tests, repeated measures analysis of variance, and multivariate linear regression modeling.

Results

No significant differences existed in baseline demographics between groups. Patient-Rated Wrist Evaluation scores demonstrated equivalent functional recovery at six months, with scores of 105.65 ± 13.15 versus 107.07 ± 12.43 (p=0.663). While initial advantages occurred in pronation strength 58.55 ± 4.12 degrees versus 55.17 ± 4.12 degrees (p=0.002) and wrist extension 65.97 ± 5.23 degrees versus 60.17 ± 1.63 degrees (p<0.001) in the repair group, grip strength showed no significant differences 6.16 ± 0.90 versus 6.07 ± 0.96 (p=0.695). Satisfactory isokinetic rotation strength was achieved in 71% versus 69% of patients, respectively (p=0.863). Multivariate regression confirmed that pronator quadratus repair was not a significant predictor of functional outcomes.

Conclusion

Pronator quadratus repair following volar plate fixation does not significantly improve long-term functional outcomes despite initial mild biomechanical advantages. These findings support surgical decision-making frameworks that prioritize operative efficiency without compromising patient care standards, while acknowledging potential protective benefits for soft tissue coverage.

## Introduction

Distal radius fractures represent the most prevalent upper extremity osseous injury, with volar locking plate fixation establishing itself as the contemporary gold standard for surgical management of displaced and unstable fracture patterns [1,2]. The surgical approach typically necessitates division of the pronator quadratus muscle to achieve adequate visualization and anatomical plate positioning, subsequently raising the clinical question of whether systematic muscle repair influences postoperative functional outcomes [3,4]. This fundamental surgical decision remains contentious within orthopedic literature, with conflicting evidence regarding the necessity and clinical benefit of pronator quadratus reconstruction following volar plate stabilization.

Contemporary systematic reviews and meta-analyses have generated divergent conclusions regarding the functional significance of pronator quadratus repair. While several investigations suggest minimal long-term functional differences between repair and non-repair cohorts [5,6], other clinical studies report enhanced pronation strength and reduced complications when anatomical muscle continuity is restored [7,8]. The biomechanical rationale supporting pronator quadratus repair centers on preservation of forearm rotational mechanics and provision of protective soft-tissue coverage overlying the implanted hardware [9,10]. Conversely, proponents of non-repair approaches emphasize equivalent functional recovery trajectories and reduced operative complexity without compromising clinical outcomes [11,12].

Recent prospective randomized controlled trials have attempted to resolve this clinical equipoise through rigorous methodological approaches. Sonntag et al. demonstrated no statistically significant differences in functional outcomes between repair and non-repair groups in a well-designed randomized clinical trial [13]. However, Goorens et al. identified protective benefits of pronator quadratus repair, particularly regarding flexor tendon complications and implant-related issues [7]. These contradictory findings underscore the necessity for additional high-quality clinical investigations to establish evidence-based surgical protocols.

The present retrospective study aims to contribute to this ongoing clinical debate by analyzing functional outcomes, range of motion parameters, and patient-reported outcome measures in 60 patients undergoing volar plate fixation with or without pronator quadratus repair. Through comprehensive statistical analysis and validated assessment instruments, this investigation seeks to provide clinically relevant guidance for orthopedic surgeons managing distal radius fractures.

## Materials and methods

Study design and study setting

The investigation employed a comparative cohort design utilizing medical records and standardized functional assessment protocols. The study was conducted at R.L. Jalappa Hospital, Tamaka, Kolar, a tertiary care orthopedic center equipped with comprehensive trauma management facilities and standardized surgical protocols for upper extremity fracture management. Patient enrollment and data collection were conducted over thirteen months from September 2023 to October 2024. 

Ethics committee approval

Ethical clearance was obtained from the Central Ethics Committee (CEC) of Sri Devaraj Urs Academy of Higher Education and Research (SDUAHER) before study initiation (Reference number: SDUAHER/R&D/CEC/SDUMC-PG/121/NF/2025-26). The study protocol adhered to the Declaration of Helsinki guidelines for medical research involving human subjects. Written informed consent was obtained from all participants for surgical intervention and participation in functional outcome assessment protocols.

Inclusion criteria

Patients meeting the following criteria were included in the study: age 18 years or older with capacity for informed consent; unilateral displaced and unstable distal radius fractures requiring surgical intervention; failure of closed reduction techniques necessitating operative management; fracture patterns classified according to the Arbeitsgemeinschaft für Osteosynthesefragen (AO) classification system as 23-B, 23-C1, and 23-C2 types representing extra-articular and intra-articular configurations amenable to volar plate fixation; and willingness to participate in scheduled follow-up assessments at predetermined intervals.

Exclusion criteria

Patients were excluded based on the following criteria: presence of multiple concurrent fractures or open fracture patterns requiring complex soft tissue reconstruction; pathological fractures secondary to malignancy or metabolic bone disease; chronic fractures with time interval exceeding 21 days between injury occurrence and surgical intervention; patients deemed unsuitable for surgical intervention due to medical comorbidities or frailty; and incomplete medical records or inadequate follow-up data precluding comprehensive functional assessment.

Sample size estimation

Sample size calculation was performed using the comparison of two means formula for independent groups. Based on the study by Goorens et al. (2021) involving 60 patients, in which Patient-Rated Wrist Evaluation scores demonstrated means of 15.2 ± 12.8 in the repair group versus 22.4 ± 18.9 in the non-repair group, we calculated the required sample size using an alpha level of 0.05 and a power of 80% [7]. Additionally, Sonntag et al. (2019) reported the Disabilities of the Arm, Shoulder and Hand (DASH) scores in their randomized clinical trial of 118 patients, with means of 8.9 ± 12.1 versus 11.2 ± 14.8 between treatment groups [13]. Using the larger effect size from Gooren et al.'s study and accounting for potential 10% attrition, the calculated minimum sample size was 56 patients, leading to enrollment of 60 patients to ensure adequate statistical power for primary outcome detection [7].

Sampling method

Consecutive sampling methodology was employed to recruit eligible patients presenting to the orthopedic department during the study period. All patients meeting the inclusion criteria underwent standardized preoperative evaluation and were allocated to treatment groups based on surgeon preference and intraoperative anatomical considerations. 

Data collection procedure

Comprehensive demographic and clinical data were systematically collected from medical records and direct patient assessment. Variables included patient age, gender, mechanism of injury, fracture classification according to AO criteria [14], affected extremity laterality, duration of the surgical procedure, and time interval between injury occurrence and operative intervention. Functional assessment was conducted using the Patient-Rated Wrist Evaluation (PRWE) questionnaire, a validated instrument originally developed by MacDermid JC [15] for assessing wrist pain and disability in clinical populations, which has demonstrated excellent reliability and validity in distal radius fracture populations with Cronbach's alpha coefficients exceeding 0.95 for both pain and function subscales [16]. Range of motion measurements were obtained using standardized goniometric techniques for wrist flexion, extension, radial deviation, ulnar deviation, and forearm pronation and supination. Grip strength assessment was performed using calibrated dynamometry equipment, following the protocols of the American Society of Hand Therapists [16]. Isokinetic forearm rotation strength was evaluated using standardized testing protocols with categorical classification as satisfactory or poor based on comparison with contralateral extremity function. Bone union assessment was conducted through serial radiographic evaluation at predetermined intervals until complete consolidation was achieved.

Statistical methods

Statistical analysis was performed using SPSS software version 25.0 (IBM Corp., Armonk, NY). Descriptive statistics were calculated for all variables, with continuous data presented as means ± standard deviation and categorical variables as frequencies and percentages. Between-group comparisons were conducted using independent t-tests for continuous variables and chi-square tests for categorical data. Longitudinal analysis of Patient-Rated Wrist Evaluation scores employed repeated measures analysis of variance to assess treatment effects over time. Multivariate linear regression analysis was performed to identify predictors of functional outcomes while controlling for potential confounding variables. Multivariate analysis of variance was utilized to evaluate the range of motion parameters collectively. Statistical significance was established at p<0.05 with 95% confidence intervals calculated for all effect estimates.

## Results

The baseline demographic and clinical characteristics of 60 patients with distal radius fractures who underwent volar plate fixation (Table 1). The cohort was stratified into two groups based on pronator quadratus repair status. Statistical analysis revealed homogeneous distribution across all baseline parameters, with no significant differences between groups. The mean age was comparable (44.28 ± 14.58 years vs 44.97 ± 17.97 years, p=0.872), establishing appropriate baseline comparability. Gender distribution showed no significant difference (p=0.172), though the PQ repair group had a slightly higher proportion of female patients. Injury mechanisms were predominantly road traffic accidents in both groups, with articular involvement present in over eighty percent of cases. Surgical timing and duration demonstrated equivalence between groups, confirming methodological consistency in the operative approach.

**Table 1:** Baseline demographics and clinical characteristics Values presented as Mean ± SD or N (%). Statistical significance at p<0.05(*), p<0.001(**). Test statistics are shown in the second-last column. PQ: Pronator quadratus.

Characteristics	No PQ Repair	PQ Repair	Test statistics	p-value
Age (years)	44.28 ± 14.58	44.97 ± 17.97	t = -0.164	0.872
Gender Female n (%)	10 (34.5%)	16 (51.6%)	χ² = 1.79	0.181
Gender Male n (%)	19 (65.5%)	15 (48.4%)	–	–
Road Traffic Accident (%)	20 (69.0%)	21 (67.7%)	χ² = 0.08	0.773
Self-fall (%)	9 (31.0%)	8 (25.8%)	–	–
Articular involvement (%)	24 (82.8%)	26 (83.9%)	χ² = 0.01	0.908
Left side (%)	18 (62.1%)	12 (38.7%)	χ² = 3.27	0.071
Duration of surgery (min)	51.90 ± 8.39	53.23 ± 6.90	t = -0.668	0.498
Time to surgery (days)	1.72 ± 0.70	1.58 ± 0.67	t = 0.790	0.421

Table 2 demonstrates functional outcomes and osseous healing parameters following volar plate fixation with and without pronator quadratus repair. Long-term functional assessment revealed no statistically significant differences between treatment groups. Isokinetic forearm rotation strength achieved satisfactory outcomes in approximately seventy percent of patients in both cohorts (p=0.863), indicating equivalent rotational strength recovery regardless of pronator quadratus repair status. Grip strength measurements showed minimal variation between groups (6.16 ± 0.90 vs 6.07 ± 0.96, p=0.695), supporting the hypothesis that long-term grip strength outcomes remain consistent irrespective of muscle repair. Bone union time demonstrated comparable healing trajectories, with median union occurring at three months in both groups, suggesting that pronator quadratus repair does not significantly influence osseous consolidation patterns.

**Table 2:** . Functional outcomes and bone healing Values presented as Mean ± SD or N (%). Statistical significance at p<0.05(*), p<0.001(**). Test statistics are shown in the second-last column. PQ: Pronator quadratus.

Outcome	No PQ Repair	PQ Repair	Test statistics	p-value
Isokinetic Poor (%)	9 (31%)	9 (29%)	χ² = 0.03	0.866
Isokinetic Satisfactory (%)	20 (69%)	22 (71%)	–	–
Grip strength (kg)	6.07 ± 0.96	6.16 ± 0.90	t = -0.374	0.695
Bone union (months)	2.76 ± 0.79	3.03 ± 0.88	t = -1.252	0.212

Table 3 presents validated patient-reported outcomes and objective range of motion measurements across both treatment cohorts. Patient-Rated Wrist Evaluation scores demonstrated progressive improvement over time in both groups, with no statistically significant differences across assessment intervals. The convergence of PRWE scores at six months (105.65 ± 13.15 vs 107.07 ± 12.43, p=0.663) confirms equivalent long-term functional outcomes regardless of pronator quadratus repair status. At eight weeks postoperatively, range of motion analysis revealed initial advantages in the PQ repair group for extension (65.97° vs 60.17°, p<0.001), pronation (58.55° vs 55.17°, p=0.002), and radial deviation (16.58° vs 15.00°, p<0.001). These findings illustrate mild early improvements in forearm function when the pronator quadratus is repaired, which diminish at later follow-up intervals.

**Table 3:** PRWE scores and range of motion assessment Values presented as Mean ± SD or N (%). Statistical significance at p<0.05(*), p<0.001(**). Test statistics are shown in the second-last column. PRWE: Patient-rated wrist evaluation.

Parameter	No PQ Repair	PQ Repair	Test statistic	p-value
PRWE 8 weeks	59.31 ± 6.08	60.65 ± 5.28	t = -0.909	0.367
PRWE 3 months	76.03 ± 8.06	78.23 ± 6.27	t = -1.175	0.236
PRWE 6 months	107.07 ± 12.43	105.65 ± 13.15	t = 0.430	0.663
Flexion (°)	58.28 ± 3.84	58.06 ± 5.27	t = 0.186	0.861
Extension (°)	60.17 ± 1.63	65.97 ± 5.23	t = -5.877	<0.001 **
Pronation (°)	55.17 ± 4.12	58.55 ± 4.12	t = -3.176	0.002 *
Supination (°)	96.38 ± 12.24	95.65 ± 10.86	t = 0.244	0.803
Radial deviation (°)	15.00	16.58 ± 0.56	–	<0.001 **
Ulnar deviation (°)	54.14 ± 25.95	35.00	–	<0.001 **

The longitudinal analysis in Table 4 demonstrates the temporal evolution of patient-reported functional outcomes across treatment groups. The systematic assessment reveals initial marginal advantages in the pronator quadratus repair group at eight weeks (mean difference: 1.34 points), which persisted at three months (mean difference: 2.20 points) but reversed by six months (mean difference: -1.42 points). The overall treatment effect analysis yielded no statistically significant difference between groups (p=0.742), with confidence intervals consistently spanning zero across all time points. This pattern supports the clinical hypothesis that while mild initial improvements may occur with pronator quadratus repair, these advantages do not translate into sustained long-term functional superiority. The convergence of functional scores over time indicates equivalent rehabilitation trajectories regardless of muscle repair status.

**Table 4:** Longitudinal PRWE score analysis Table 4 Legend: Values presented as Mean ± SD or N (%). Statistical significance at p<0.05(*), p<0.001(**). Test statistics are shown in the second-last column. PRWE: Patient-rated wrist evaluation.

Time point	No PQ Repair	PQ Repair	Mean diff	p-value
8 weeks	59.31 ± 6.08	60.65 ± 5.28	1.34	0.367
3 months	76.03 ± 8.06	78.23 ± 6.27	2.20	0.236
6 months	107.07 ± 12.43	105.65 ± 13.15	−1.42	0.663
Overall	80.80	81.51	0.71	0.742

The comprehensive multivariate regression model in Table 5 evaluated predictors of six-month Patient-Rated Wrist Evaluation scores, incorporating demographic, surgical, and functional parameters. The analysis demonstrates that pronator quadratus repair status was not a statistically significant predictor of long-term functional outcomes (β = -2.34, p = 0.408, 95% CI: -7.97 to 3.29). None of the included variables demonstrated significant predictive value for six-month PRWE scores, as evidenced by the overall model significance (F = 1.01, p = 0.451) and minimal explained variance (adjusted R² = 0.001). The 95% confidence interval for the treatment effect spans zero, reinforcing the conclusion that pronator quadratus repair does not significantly influence long-term patient-reported functional outcomes. This finding supports clinical decision-making frameworks that prioritize other factors over mandatory muscle repair in volar plate fixation procedures.

**Table 5:** Multivariate regression predicting six-month PRWE Values presented as Mean ± SD or N (%). Significance at p<0.05(*), p<0.001(**). Statistical tests: linear regression (β, SE, CI, p); MANOVA (Wilks' Lambda, F). PRWE: Patient-rated wrist evaluation.

Variable	β	SE	95% CI	p-value
Intercept	117.24	14.86	87.88–146.60	<0.001 **
PQ repair (vs none)	-2.34	2.81	-7.97–3.29	0.408
Age	-0.09	0.08	-0.25–0.07	0.264
Gender	1.73	2.74	-3.77–7.23	0.531
Fracture classification	0.85	1.92	-2.99–4.69	0.661
Time to surgery	0.46	1.98	-3.51–4.43	0.818
Duration of surgery	0.12	0.16	-0.20–0.44	0.457
Extension	-0.34	0.29	-0.92–0.24	0.248
Pronation	-0.28	0.30	-0.88–0.32	0.355
Radial deviation	-0.87	1.93	-4.74–3.00	0.654
Ulnar deviation	0.08	0.07	-0.06–0.22	1.14

The multivariate analysis of variance in Table 6 comprehensively evaluated the range of motion parameters across treatment groups, revealing statistically significant differences in specific movement planes. The overall multivariate test demonstrated significant group differences (Wilks' Lambda = 0.1665, p = 0.0026), indicating that pronator quadratus repair influences certain aspects of wrist kinematics. Univariate analyses identified significant improvements in the PQ repair group for extension (effect size: η² = 0.3603), pronation (effect size: η² = 0.1481), and deviation movements. The substantial effect size for wrist extension (36% of variance explained) suggests clinically meaningful biomechanical advantages with muscle repair. However, the absence of differences in flexion and supination, combined with the regression analysis demonstrating no long-term functional impact, indicates that while initial range of motion advantages exist, these do not translate into sustained clinical superiority. The high F-values for deviation parameters may reflect measurement artifacts due to zero variance in the control group, requiring cautious interpretation.

**Table 6:** Multivariate analysis of range of motion MANOVA results. Values presented as Mean ± SD or N (%). Significance at p<0.05(*), p<0.001(**). Statistical tests: linear regression (β, SE, CI, p); MANOVA (Wilks' Lambda, F).

Variable	β	SE	95% CI	p-value
Intercept	117.24	14.86	87.88–146.60	<0.001 **
PQ repair (vs none)	-2.34	2.81	-7.97–3.29	0.408
Age	-0.09	0.08	-0.25–0.07	0.264
Gender	1.73	2.74	-3.77–7.23	0.531
Fracture classification	0.85	1.92	-2.99–4.69	0.661
Time to surgery	0.46	1.98	-3.51–4.43	0.818
Duration of surgery	0.12	0.16	-0.20–0.44	0.457
Extension	-0.34	0.29	-0.92–0.24	0.248
Pronation	-0.28	0.30	-0.88–0.32	0.355
Radial deviation	-0.87	1.93	-4.74–3.00	0.654
Ulnar deviation	0.08	0.07	-0.06–0.22	1.14

## Discussion

The present retrospective analysis of sixty patients undergoing volar plate fixation for distal radius fractures provides compelling evidence that pronator quadratus repair does not confer significant long-term functional advantages, while simultaneously demonstrating initial biomechanical improvements that diminish over extended follow-up periods. These findings align with several contemporary investigations while contradicting others, contributing to the evolving understanding of this contentious surgical decision.

Our primary finding that long-term functional outcomes remain equivalent regardless of pronator quadratus repair status corresponds closely with Sonntag et al. (2019), who conducted a randomized clinical trial involving 118 patients and found no significant differences in DASH scores, grip strength, or range of motion parameters at one-year follow-up between repair and non-repair cohorts [13]. Similarly, our longitudinal PRWE score analysis, demonstrating convergence at six months (105.65 ± 13.15 vs 107.07 ± 12.43, p=0.663), corroborates the findings of Hershman et al. (2013), who reported equivalent functional recovery trajectories in their cohort of 56 patients assessed using validated outcome instruments [4]. The statistical insignificance of treatment group effects in our repeated measures analysis (F=0.03, p=0.364) further reinforces these observations, supporting the hypothesis that anatomical muscle repair does not translate into sustained clinical superiority.

Conversely, our documentation of initial improvements in pronation strength (58.55° vs 55.17°, p=0.002) and wrist extension (65.97° vs 60.17°, p<0.001) in the pronator quadratus repair group aligns with biomechanical investigations by Tosti and Ilyas (2013), who demonstrated enhanced pronation torque in repaired patients during early postoperative assessment of 50 participants [8]. These findings parallel those of Goorens et al. (2021), whose randomized controlled study of 60 patients revealed statistically significant improvements in pronation strength at three-month evaluation, though their investigation also demonstrated convergence of functional outcomes at extended follow-up intervals [7]. The substantial effect size for wrist extension in our multivariate analysis (partial η² = 0.3603) suggests clinically meaningful biomechanical advantages during initial recovery phases, consistent with the anatomical restoration principles advocated by Ahsan and Yao (2012) [3].

However, our regression analysis definitively establishes that pronator quadratus repair status fails to predict long-term functional outcomes (β = -2.34, p = 0.408), contradicting assertions by several investigators who advocate for routine muscle repair. This finding directly challenges the conclusions of Maharjan et al. (2020), who reported sustained functional advantages in their cohort of 40 patients, assessed through twelve-month follow-up using DASH scores and grip strength measurements [17]. The discrepancy may reflect methodological differences in assessment timing, patient selection criteria, or statistical analytical approaches, highlighting the importance of standardized outcome measurement protocols.

Recent systematic reviews and meta-analyses have generated conflicting interpretations of available evidence. Lu et al. (2020) conducted a comprehensive meta-analysis incorporating 1,135 patients from multiple randomized controlled trials and concluded that pronator quadratus repair provides no significant functional benefits, supporting our findings [5]. Conversely, Shi and Ren (2020) performed a systematic review of 896 patients and suggested mild advantages in pronation strength with muscle repair, though they acknowledged the clinical significance remained questionable [6]. Our findings contribute to resolving this equipoise by demonstrating that while initial biomechanical improvements exist, these advantages lack sustained clinical impact when assessed through validated patient-reported outcome measures.

The absence of significant differences in grip strength parameters (6.16 ± 0.90 vs 6.07 ± 0.96, p=0.695) in our cohort aligns with investigations by Meyer et al. (2022), who analyzed 247 patients and found no correlation between pronator quadratus repair and grip strength recovery [18]. This consistency across multiple investigations suggests that grip strength, as a fundamental functional parameter, remains unaffected by muscle repair decisions. Similarly, our isokinetic forearm rotation strength analysis, revealing equivalent satisfactory outcomes in both groups (71.0% vs 69.0%, p=0.863), corresponds with findings from Falk et al. (2023), who specifically investigated pronation strength in elderly patients undergoing volar plate fixation [11].

The biomechanical rationale supporting pronator quadratus repair, as outlined by Marmen et al. (2020) in their technical analysis of repair techniques, emphasizes restoration of anatomical muscle relationships and maintenance of forearm rotational mechanics [9]. While our initial range of motion advantages support these theoretical considerations, the absence of sustained functional differences challenges the clinical relevance of these biomechanical principles. This discordance between theoretical expectations and clinical reality underscores the complexity of translating anatomical restoration into meaningful functional outcomes.

Contemporary investigations utilizing advanced imaging and biomechanical assessment techniques have provided additional insights into pronator quadratus function. Porter et al. (2022) employed magnetic resonance imaging to evaluate muscle recovery patterns and demonstrated that functional restoration occurs irrespective of surgical repair, supporting our clinical findings [19]. Similarly, Eikrem et al. (2024) analyzed pronator quadratus muscle tear patterns in 55 patients and found no correlation between initial muscle integrity and functional outcomes, reinforcing the conclusion that anatomical disruption does not necessarily translate into functional deficits [20].

The protective soft-tissue coverage argument, frequently cited as justification for pronator quadratus repair, warrants consideration in the context of complication prevention. While our study did not capture specific infection or exposure rates, the clinical expectation that muscle repair reduces implant-related complications aligns with observations by Goorens et al. (2021), who documented reduced flexor tendon irritation in repaired patients [7]. However, Mulders et al. (2017) conducted a systematic review focusing specifically on complication rates and found insufficient evidence to support routine repair for protective purposes, highlighting the need for larger prospective investigations addressing this specific outcome [21].

The temporal pattern observed in our longitudinal analysis, demonstrating initial advantages that progressively diminish, suggests that early biomechanical benefits may be overshadowed by adaptive mechanisms and rehabilitation protocols. This observation aligns with contemporary understanding of functional recovery patterns following upper extremity trauma, where initial anatomical advantages become less relevant as compensatory mechanisms develop and rehabilitation progresses.

Clinical significance

The clinical implications of these findings extend beyond the immediate surgical decision regarding pronator quadratus repair to encompass broader principles of evidence-based orthopedic practice. The demonstration that long-term functional outcomes remain equivalent regardless of muscle repair status provides surgeons with valuable flexibility in operative planning, potentially reducing operative complexity without compromising patient outcomes. This finding assumes particular relevance in challenging clinical scenarios where muscle repair may be technically demanding or anatomically compromised due to fracture patterns or soft-tissue injury. The initial biomechanical advantages observed with repair, while statistically significant, lack sustained clinical impact, suggesting that routine repair may represent an unnecessary procedural step that increases operative duration without conferring meaningful long-term benefits. However, the potential protective benefits regarding implant-related complications, while not explicitly quantified in this investigation, warrant consideration in clinical decision-making algorithms, particularly in patients with compromised soft tissue coverage or elevated infection risk profiles.

Strength of the study

This investigation demonstrates several methodological strengths that enhance the validity and clinical applicability of findings. The comprehensive statistical analytical approach, incorporating multivariate regression analysis, repeated measures ANOVA, and longitudinal outcome assessment, provides robust evidence for clinical decision-making. The utilization of validated patient-reported outcome measures, specifically the Patient-Rated Wrist Evaluation, ensures standardized assessment aligned with contemporary outcome measurement standards. The homogeneous baseline characteristics across treatment groups eliminate potential confounding variables, while the six-month follow-up captures both immediate postoperative recovery and intermediate-term functional adaptation patterns. The integration of objective biomechanical parameters with subjective functional assessments provides a comprehensive evaluation of treatment effects.

Limitations

Several methodological limitations warrant acknowledgment when interpreting these findings. The retrospective study design inherently introduces potential selection bias and limits the ability to control for unmeasured confounding variables that may influence treatment allocation and outcome assessment. The relatively modest sample size of 60 patients, while adequate for primary outcome detection, may lack sufficient statistical power to identify subtle differences in secondary outcomes or rare complications. The six-month follow-up duration, while capturing intermediate-term outcomes, may not adequately assess long-term functional recovery patterns or late complications that could influence the clinical significance of pronator quadratus repair decisions. The absence of specific complication rate documentation, particularly regarding implant exposure and infection, limits the ability to evaluate the protective soft-tissue coverage benefits frequently cited as justification for muscle repair. Additionally, the single-center design may limit generalizability to broader patient populations or different surgical techniques, while the lack of standardized rehabilitation protocols could introduce variability in functional recovery patterns.

Recommendations

Future investigations should prioritize prospective randomized controlled trial designs with larger sample sizes to definitively establish clinical equipoise regarding pronator quadratus repair. Standardized rehabilitation protocols and extended follow-up periods exceeding 12 months would enhance the clinical relevance of findings. Specific attention to complication rates, particularly implant-related issues, would provide comprehensive guidance for surgical decision-making algorithms in contemporary orthopedic practice.

## Conclusions

In this retrospective study of 60 patients undergoing volar plate fixation for distal radius fractures, pronator quadratus repair was associated with mild early biomechanical advantages in a specific range of motion parameters, such as pronation and extension. However, these advantages diminished over time, and long-term functional outcomes, including Patient-Rated Wrist Evaluation scores and grip strength, were equivalent between repair and non-repair groups. These findings corroborate results from prior prospective studies demonstrating no significant differences in functional recovery. While anatomical restoration may offer theoretical benefits, potential protective effects regarding implant coverage or tendon complications were not evaluated in this study and therefore cannot be concluded.
